# Interdisciplinary education group in type 1 diabetes

**DOI:** 10.1186/1758-5996-7-S1-A232

**Published:** 2015-11-11

**Authors:** Lucia Hitomi Kamata Lopes, Cleber Aparecido dos Santos, Mariana Ercole Bauléo, Monica de Cassia Brigeiro Martinez

**Affiliations:** 1 Hospital Israelita Albert Einstein, São Paulo, Brazil

## Background

Diabetes mellitus type 1 (DM1) is an autoimmune disease that manifests in childhood or adolescence, promoted by progressive immune-mediated destruction of pancreatic beta cells, resulting in absolute insulin deficiency in the body, which makes patients dependent on insulin. The complexity of the treatment that includes: medication adjustments, nutrition education, blood glucose monitoring and changes in lifestyle habits, factors that lead to difficulty in adherence proposal. The diagnosis affects not only the child but the whole family structure, with impact on the day-to-day and emotional and affective sphere. Education is fundamental to motivate the patient to acquire knowledge and skills promoting self-management of DM1 care generating changes in behavior and better quality of life.

## Objective

To provide better adherence to treatment, disease control and better quality of life by reducing the risk of complications.

## Materials and methods

Monthly educational meetings at a clinic for pediatric specialties, reference to disadvantaged communities of São Paulo, aimed at children with DM1 and their families. The education group in diabetes happens through group education, interactive group, educational games, cooking classes, Practice staff training patients to self-administer insulin, and self-blood glucose monitoring, physical and recreational activities with professionals from an interdisciplinary team (endocrinologist, nutritionist, gastronomy, nurse, psychologist, pharmacist, physical educator and volunteer).

## Results

Reports of children and their families show that the group not only contributes to the control of the disease but makes a difference in the life of each. “I learned to give more freedom my daughter and so she learned to take care, to apply insulin, have more confidence, be more independent.” “I learned to share what I know with others and learned from them too.” “In addition to taking questions, it helps to let off steam and have more confidence.”

## Conclusion

A multidisciplinary approach, using unconventional therapeutic resources (educational and recreational), if It shows extremely important in diabetes education as a way to increase awareness and acceptance of the disease, through exchanges of experience, technical skills and involvement in decision-making in relation to self-care.

